# Supplementary material for: A yeast phenomic model for the influence of Warburg metabolism on genetic buffering of doxorubicin
Source: Cancer Metab. 2019 Oct 23;7:9. doi: 10.1186/s40170-019-0201-3 (PMC6806529; doi:10.1186/s40170-019-0201-3)
Supplement: Supplementary file 9 — Additional file 9. GO term-specific heatmaps for REMc/GTF-enriched clusters. GO term-specific heatmaps for significant GO process terms were generated as described in methods and Figs. 3 and 4. Any related child terms are presented in subsequent pages of the parent file name. GO terms with more than 100 children, with 2 or fewer genes annotated to the term, or a file size over 300KB are not shown. All heatmaps are generated with the same layout (see Figs. 3 and 4). [file 40170_2019_201_MOESM9_ESM.bz2 › Additional_File9_GOTermHeatmaps/Additional_File9_GOTermHeatmap/RNA_phosphodiester_bond_hydrolysis.pdf]

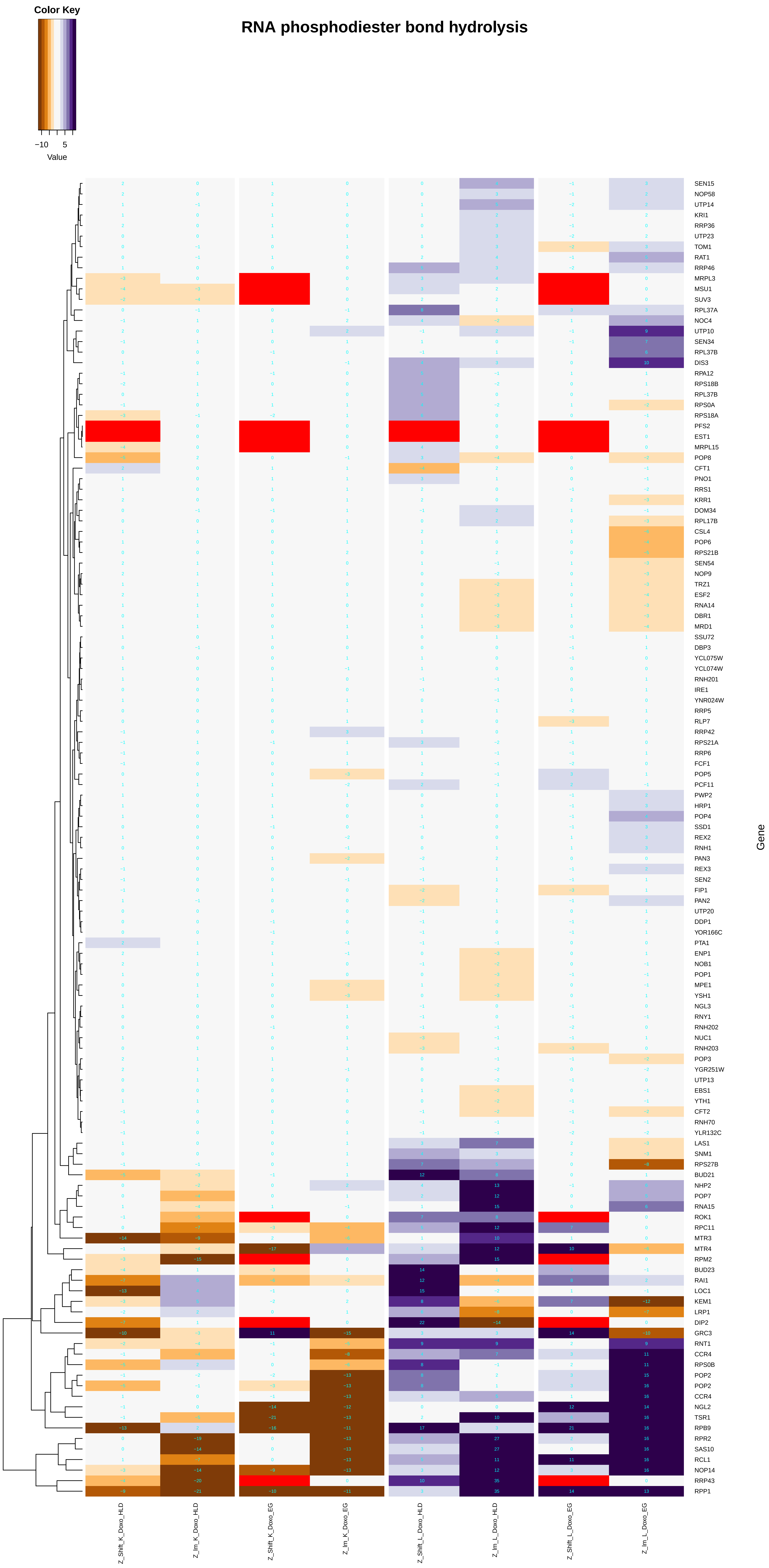
Color Key
−10
5
Value
RNA phosphodiester bond hydrolysis
SEN15
NOP58
UTP14
KRI1
RRP36
UTP23
TOM1
RAT1
RRP46
MRPL3
MSU1
SUV3
RPL37A
NOC4
UTP10
SEN34
RPL37B
DIS3
RPA12
RPS18B
RPL37B
RPS0A
RPS18A
PFS2
EST1
MRPL15
POP8
CFT1
PNO1
RRS1
KRR1
DOM34
RPL17B
CSL4
POP6
RPS21B
SEN54
NOP9
TRZ1
ESF2
RNA14
DBR1
MRD1
SSU72
DBP3
YCL075W
YCL074W
RNH201
IRE1
YNR024W
RRP5
RLP7
RRP42
RPS21A
RRP6
FCF1
POP5
PCF11
PWP2
HRP1
POP4
SSD1
REX2
RNH1
PAN3
REX3
SEN2
FIP1
PAN2
UTP20
DDP1
YOR166C
PTA1
ENP1
NOB1
POP1
MPE1
YSH1
NGL3
RNY1
RNH202
NUC1
RNH203
POP3
YGR251W
UTP13
EBS1
YTH1
CFT2
RNH70
YLR132C
LAS1
SNM1
RPS27B
BUD21
NHP2
POP7
RNA15
ROK1
RPC11
MTR3
MTR4
RPM2
BUD23
RAI1
LOC1
KEM1
LRP1
DIP2
GRC3
RNT1
CCR4
RPS0B
POP2
POP2
CCR4
NGL2
TSR1
RPB9
RPR2
SAS10
RCL1
NOP14
RRP43
RPP1
Gene
Z_Shift_K_Doxo_HLD
Z_Im_K_Doxo_HLD
Z_Shift_K_Doxo_EG
Z_Im_K_Doxo_EG
Z_Shift_L_Doxo_HLD
Z_Im_L_Doxo_HLD
Z_Shift_L_Doxo_EG
Z_Im_L_Doxo_EG

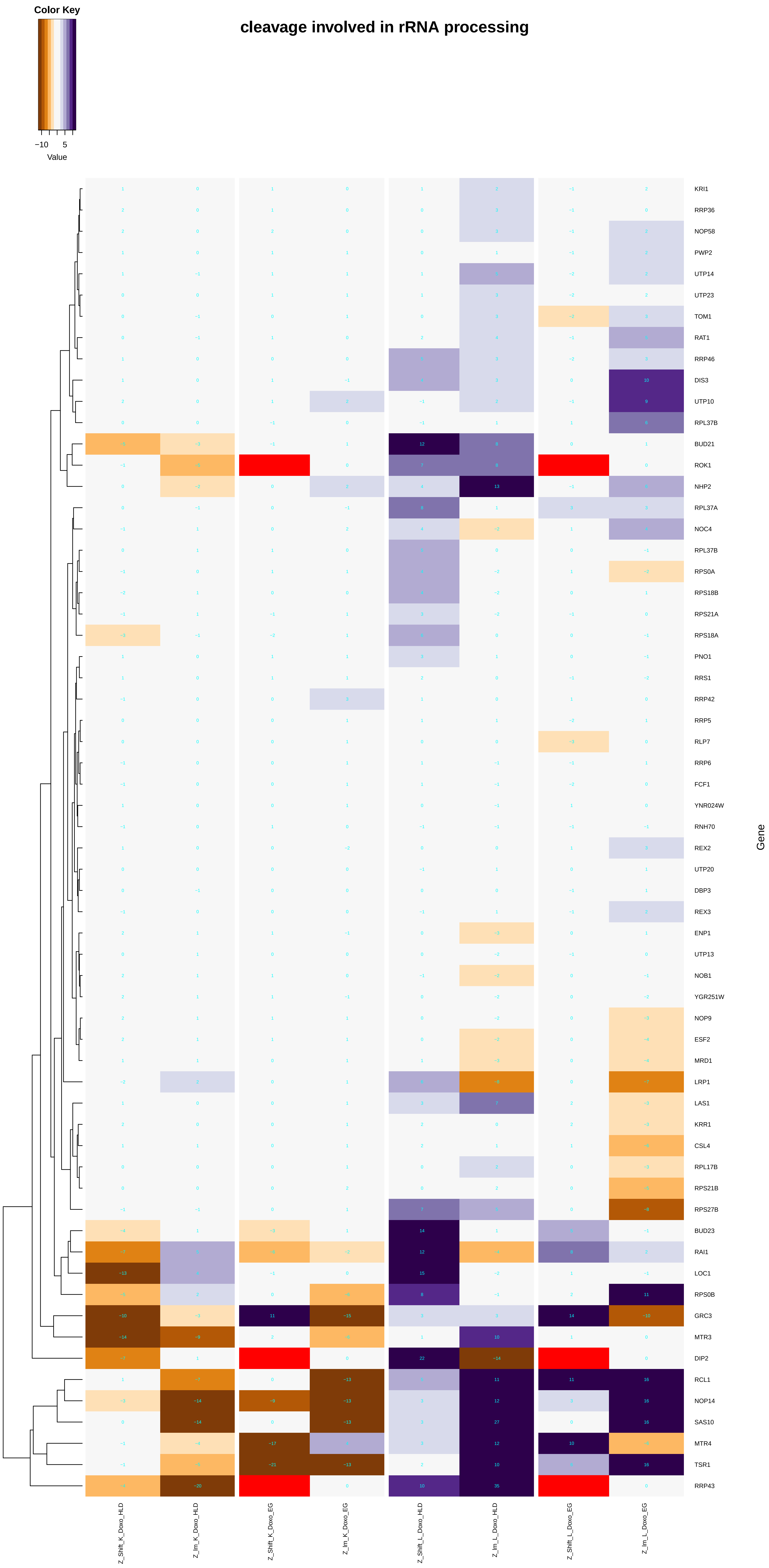

Color Key
−10
5
Value
cleavage involved in rRNA processing
KRI1
RRP36
NOP58
PWP2
UTP14
UTP23
TOM1
RAT1
RRP46
DIS3
UTP10
RPL37B
BUD21
ROK1
NHP2
RPL37A
NOC4
RPL37B
RPS0A
RPS18B
RPS21A
RPS18A
PNO1
RRS1
RRP42
RRP5
RLP7
RRP6
FCF1
YNR024W
RNH70
REX2
UTP20
DBP3
REX3
ENP1
UTP13
NOB1
YGR251W
NOP9
ESF2
MRD1
LRP1
LAS1
KRR1
CSL4
RPL17B
RPS21B
RPS27B
BUD23
RAI1
LOC1
RPS0B
GRC3
MTR3
DIP2
RCL1
NOP14
SAS10
MTR4
TSR1
RRP43
Gene
Z_Shift_K_Doxo_HLD
Z_Im_K_Doxo_HLD
Z_Shift_K_Doxo_EG
Z_Im_K_Doxo_EG
Z_Shift_L_Doxo_HLD
Z_Im_L_Doxo_HLD
Z_Shift_L_Doxo_EG
Z_Im_L_Doxo_EG

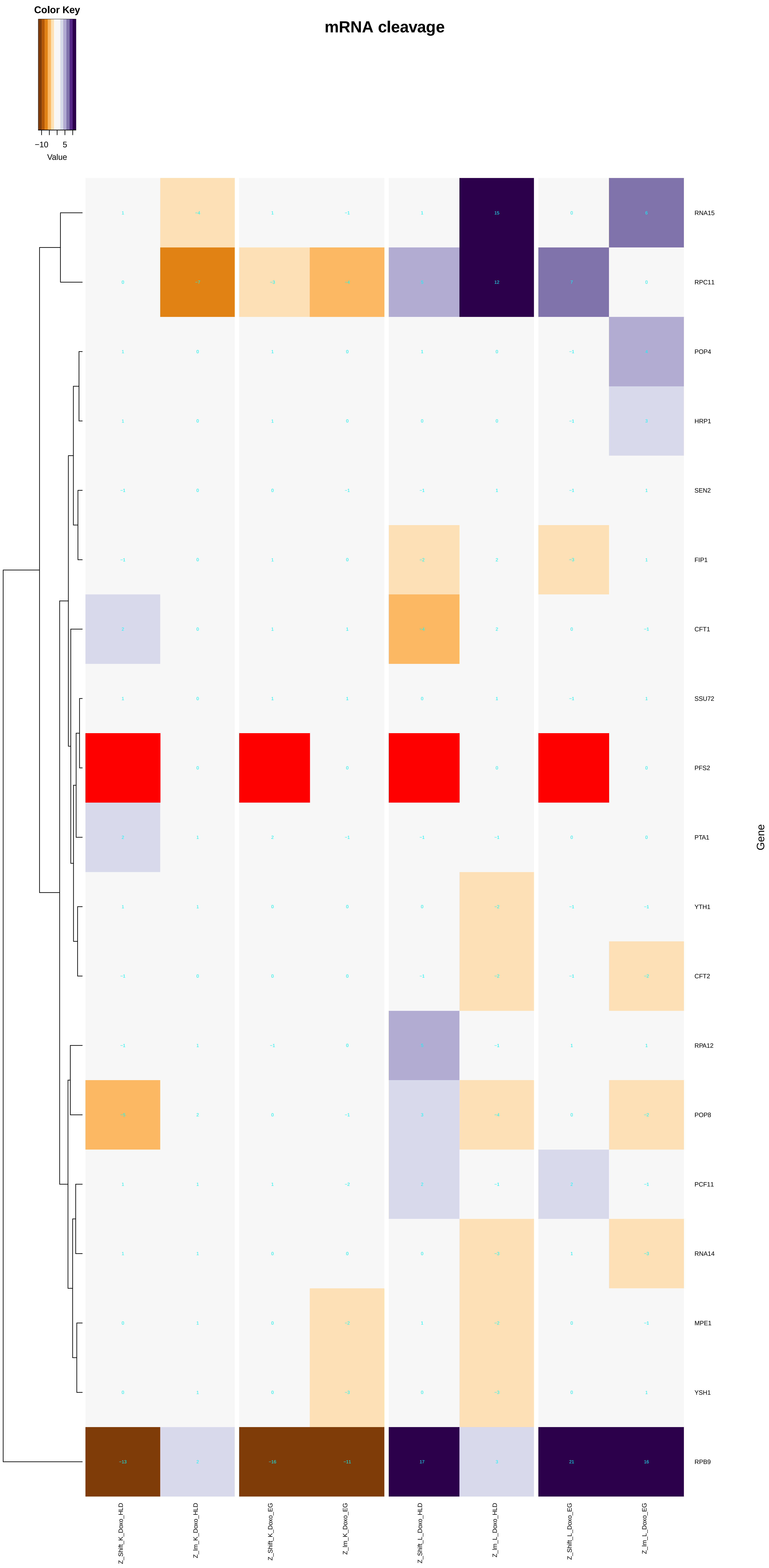

Color Key
−10
5
Value
mRNA cleavage
RNA15
RPC11
POP4
HRP1
SEN2
FIP1
CFT1
SSU72
PFS2
PTA1
YTH1
CFT2
RPA12
POP8
PCF11
RNA14
MPE1
YSH1
RPB9
Gene
Z_Shift_K_Doxo_HLD
Z_Im_K_Doxo_HLD
Z_Shift_K_Doxo_EG
Z_Im_K_Doxo_EG
Z_Shift_L_Doxo_HLD
Z_Im_L_Doxo_HLD
Z_Shift_L_Doxo_EG
Z_Im_L_Doxo_EG

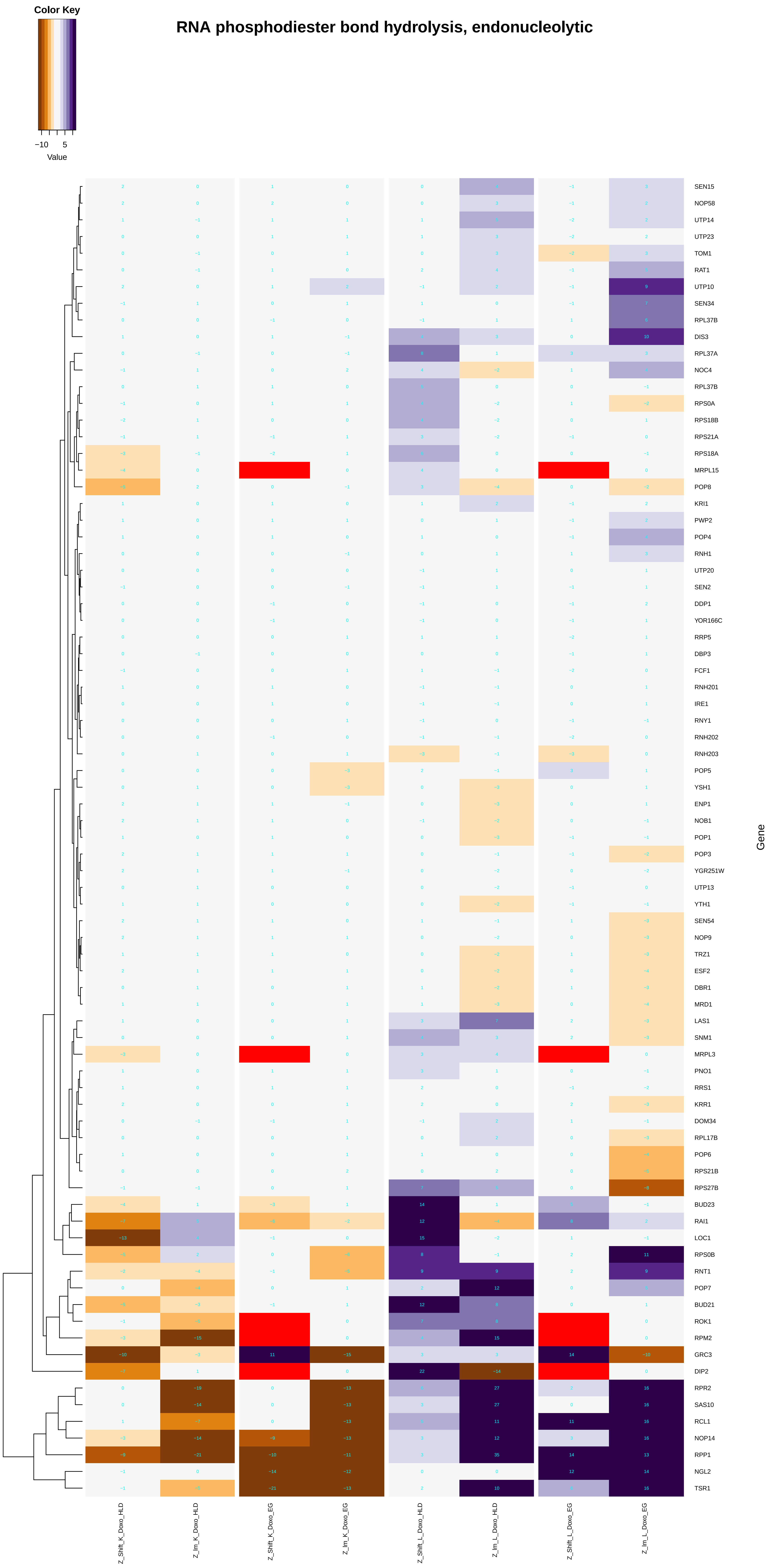
Color Key
−10
5
Value
RNA phosphodiester bond hydrolysis, endonucleolytic
SEN15
NOP58
UTP14
UTP23
TOM1
RAT1
UTP10
SEN34
RPL37B
DIS3
RPL37A
NOC4
RPL37B
RPS0A
RPS18B
RPS21A
RPS18A
MRPL15
POP8
KRI1
PWP2
POP4
RNH1
UTP20
SEN2
DDP1
YOR166C
RRP5
DBP3
FCF1
RNH201
IRE1
RNY1
RNH202
RNH203
POP5
YSH1
ENP1
NOB1
POP1
POP3
YGR251W
UTP13
YTH1
SEN54
NOP9
TRZ1
ESF2
DBR1
MRD1
LAS1
SNM1
MRPL3
PNO1
RRS1
KRR1
DOM34
RPL17B
POP6
RPS21B
RPS27B
BUD23
RAI1
LOC1
RPS0B
RNT1
POP7
BUD21
ROK1
RPM2
GRC3
DIP2
RPR2
SAS10
RCL1
NOP14
RPP1
NGL2
TSR1
Gene
Z_Shift_K_Doxo_HLD
Z_lm_K_Doxo_HLD
Z_Shift_K_Doxo_EG
Z_lm_K_Doxo_EG
Z_Shift_L_Doxo_HLD
Z_lm_L_Doxo_HLD
Z_Shift_L_Doxo_EG
Z_lm_L_Doxo_EG

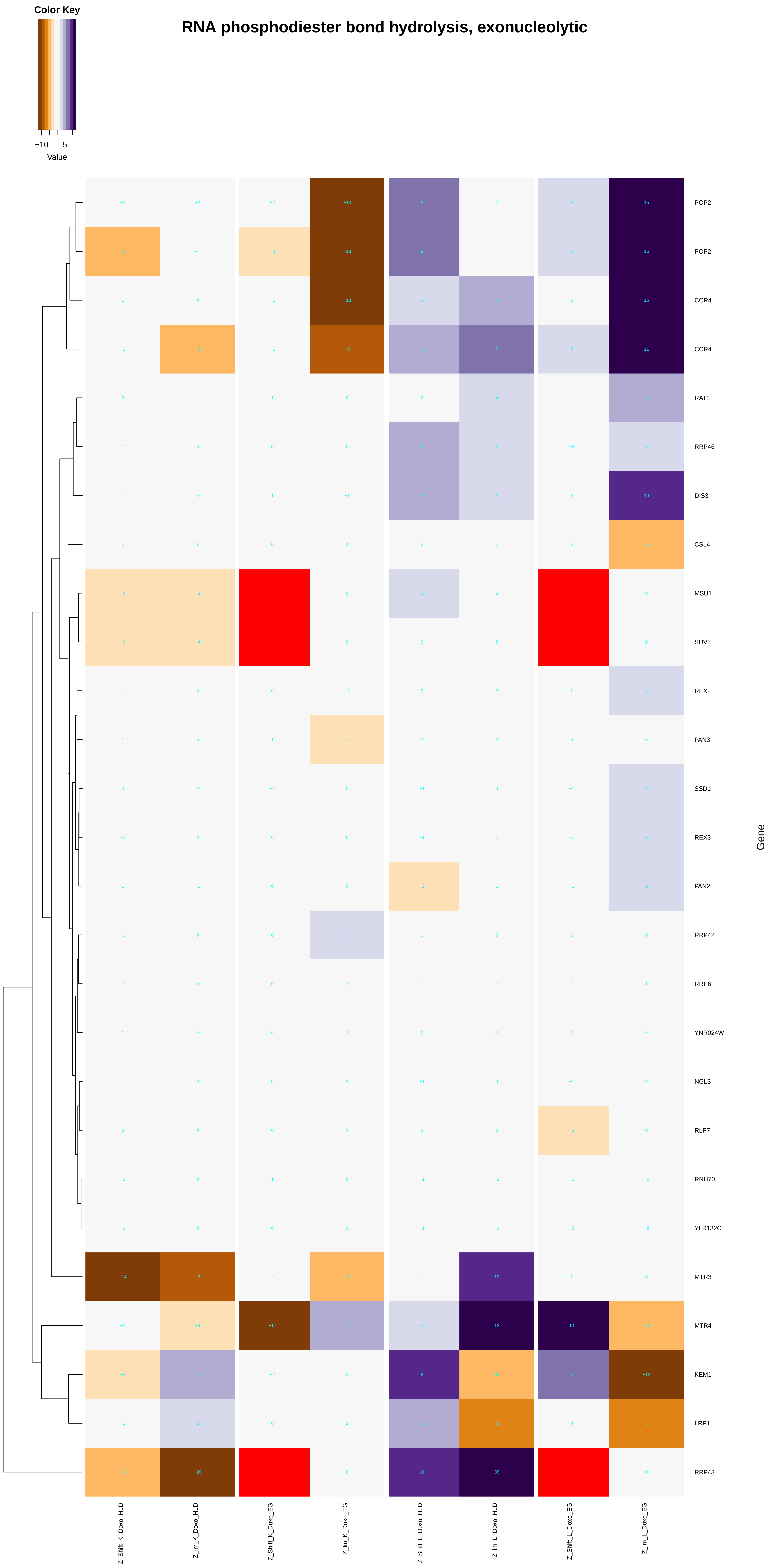

RNA phosphodiester bond hydrolysis, exonucleolytic
Color Key
−10
5
Value
POP2
POP2
CCR4
CCR4
RAT1
RRP46
DIS3
CSL4
MSU1
SUV3
REX2
PAN3
SSD1
REX3
PAN2
RRP42
RRP6
YNR024W
NGL3
RLP7
RNH70
YLR132C
MTR3
MTR4
KEM1
LRP1
RRP43
Gene
Z_Shift_K_Doxo_HLD
Z_lm_K_Doxo_HLD
Z_Shift_K_Doxo_EG
Z_lm_K_Doxo_EG
Z_Shift_L_Doxo_HLD
Z_lm_L_Doxo_HLD
Z_Shift_L_Doxo_EG
Z_lm_L_Doxo_EG

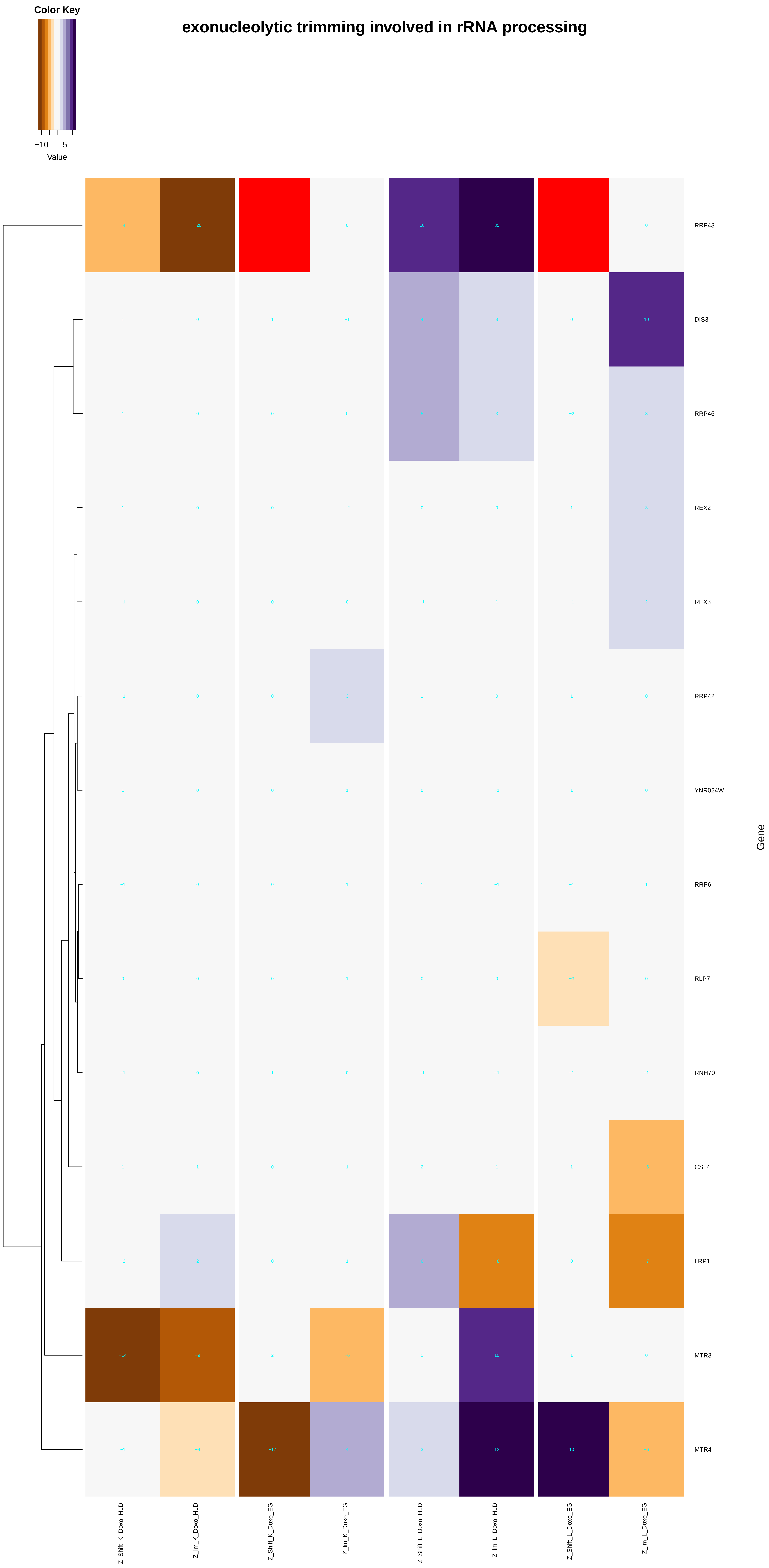
Color Key
−10
5
Value
exonucleolytic trimming involved in rRNA processing
RRP43
DIS3
RRP46
REX2
REX3
RRP42
YNR024W
RRP6
RLP7
RNH70
CSL4
LRP1
MTR3
MTR4
Gene
Z_Shift_K_Doxo_HLD
Z_lm_K_Doxo_HLD
Z_Shift_K_Doxo_EG
Z_lm_K_Doxo_EG
Z_Shift_L_Doxo_HLD
Z_lm_L_Doxo_HLD
Z_Shift_L_Doxo_EG
Z_lm_L_Doxo_EG

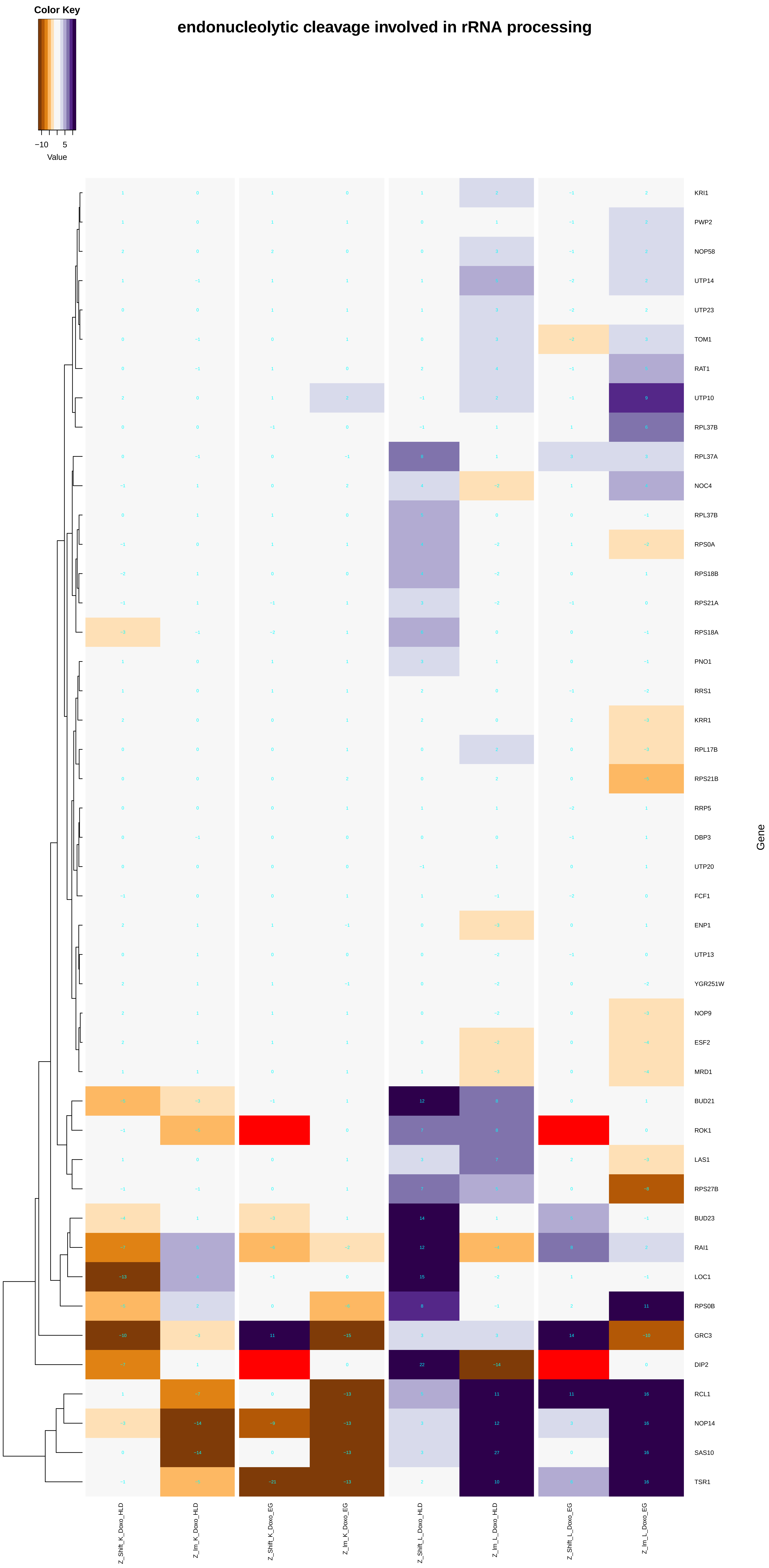
Color Key
−10 5
Value
endonucleolytic cleavage involved in rRNA processing
KRI1
PWP2
NOP58
UTP14
UTP23
TOM1
RAT1
UTP10
RPL37B
RPL37A
NOC4
RPL37B
RPS0A
RPS18B
RPS21A
RPS18A
PNO1
RRS1
KRR1
RPL17B
RPS21B
RRP5
DBP3
UTP20
FCF1
ENP1
UTP13
YGR251W
NOP9
ESF2
MRD1
BUD21
ROK1
LAS1
RPS27B
BUD23
RAI1
LOC1
RPS0B
GRC3
DIP2
RCL1
NOP14
SAS10
TSR1
Gene
Z_Shift_K_Doxo_HLD
Z_lm_K_Doxo_HLD
Z_Shift_K_Doxo_EG
Z_lm_K_Doxo_EG
Z_Shift_L_Doxo_HLD
Z_lm_L_Doxo_HLD
Z_Shift_L_Doxo_EG
Z_lm_L_Doxo_EG

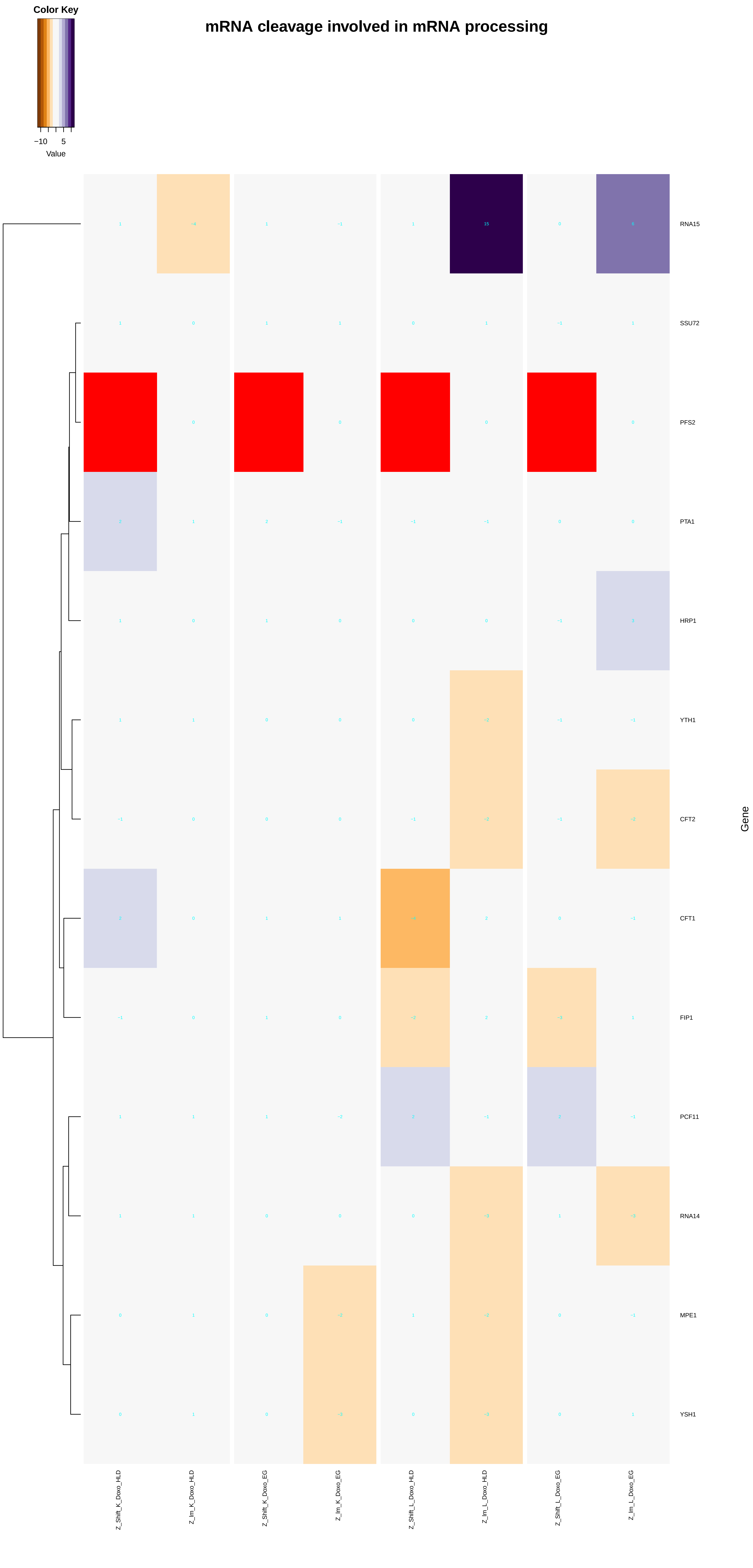
Color Key
−10
5
Value
mRNA cleavage involved in mRNA processing
RNA15
SSU72
PFS2
PTA1
HRP1
YTH1
CFT2
CFT1
FIP1
PCF11
RNA14
MPE1
YSH1
Gene
Z_Shift_K_Doxo_HLD
Z_Im_K_Doxo_HLD
Z_Shift_K_Doxo_EG
Z_Im_K_Doxo_EG
Z_Shift_L_Doxo_HLD
Z_Im_L_Doxo_HLD
Z_Shift_L_Doxo_EG
Z_Im_L_Doxo_EG

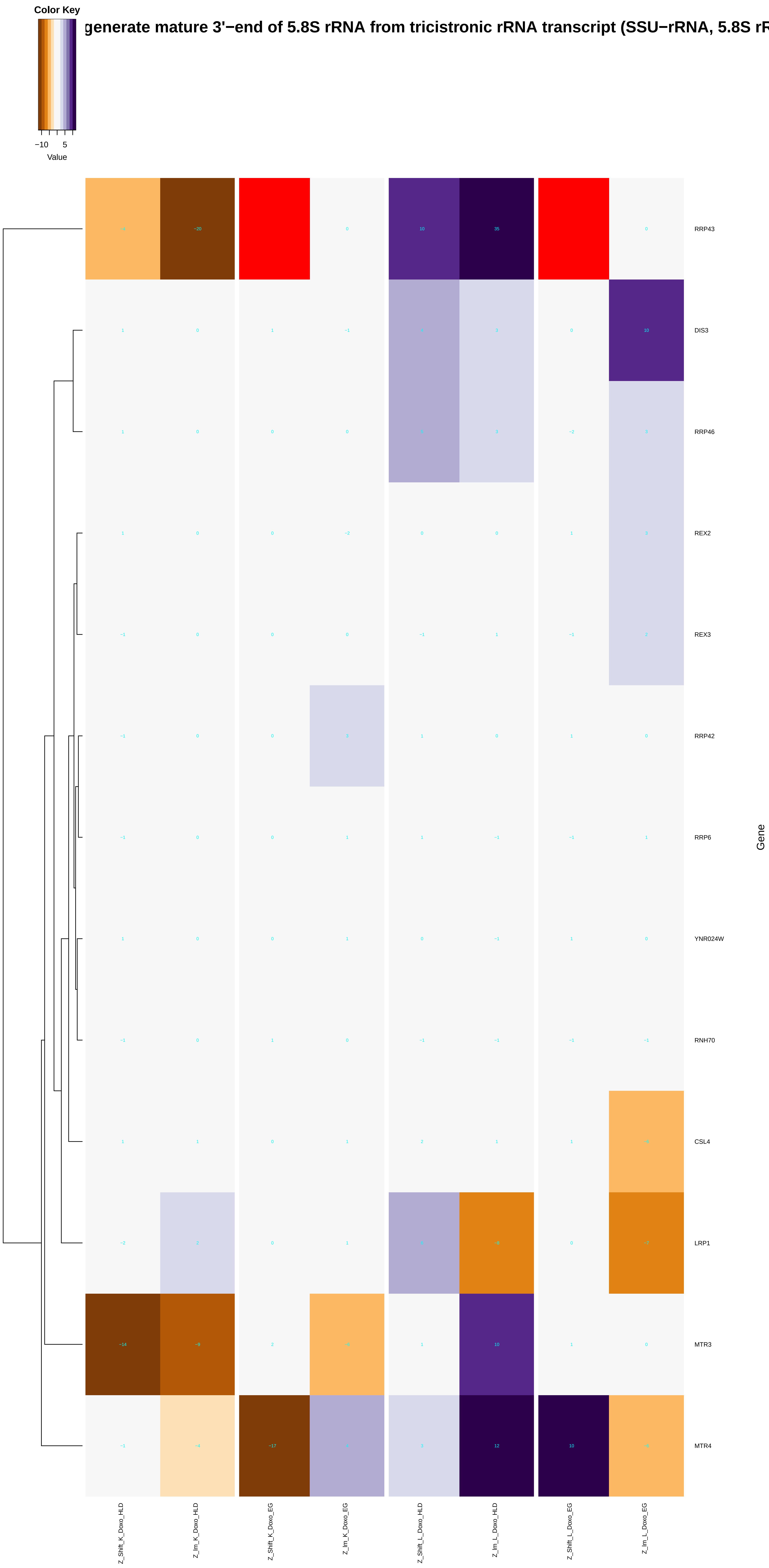

Color Key
−10
5
Value
generate mature 3'−end of 5.8S rRNA from tricistronic rRNA transcript (SSU−rRNA, 5.8S rR
RRP43
DIS3
RRP46
REX2
REX3
RRP42
RRP6
YNR024W
RNH70
CSL4
LRP1
MTR3
MTR4
Gene
Z_Shift_K_Doxo_HLD
Z_Im_K_Doxo_HLD
Z_Shift_K_Doxo_EG
Z_Im_K_Doxo_EG
Z_Shift_L_Doxo_HLD
Z_Im_L_Doxo_HLD
Z_Shift_L_Doxo_EG
Z_Im_L_Doxo_EG

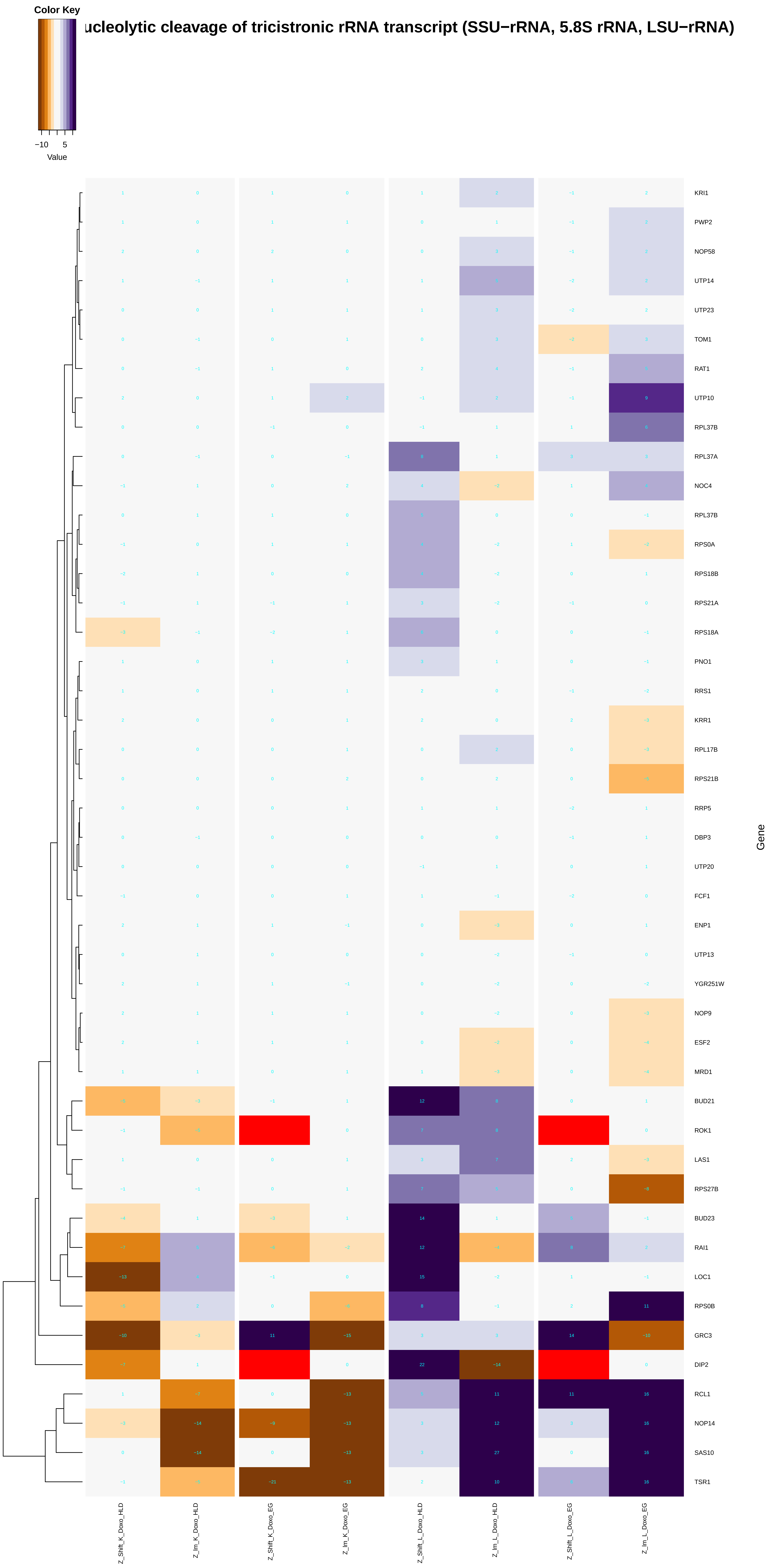

Color Key
−10 5
Value
ucleolytic cleavage of tricistronic rRNA transcript (SSU−rRNA, 5.8S rRNA, LSU−rRNA)
KRI1
PWP2
NOP58
UTP14
UTP23
TOM1
RAT1
UTP10
RPL37B
RPL37A
NOC4
RPL37B
RPS0A
RPS18B
RPS21A
RPS18A
PNO1
RRS1
KRR1
RPL17B
RPS21B
RRP5
DBP3
UTP20
FCF1
ENP1
UTP13
YGR251W
NOP9
ESF2
MRD1
BUD21
ROK1
LAS1
RPS27B
BUD23
RAI1
LOC1
RPS0B
GRC3
DIP2
RCL1
NOP14
SAS10
TSR1
Gene
Z_Shift_K_Doxo_HLD
Z_Im_K_Doxo_HLD
Z_Shift_K_Doxo_EG
Z_Im_K_Doxo_EG
Z_Shift_L_Doxo_HLD
Z_Im_L_Doxo_HLD
Z_Shift_L_Doxo_EG
Z_Im_L_Doxo_EG

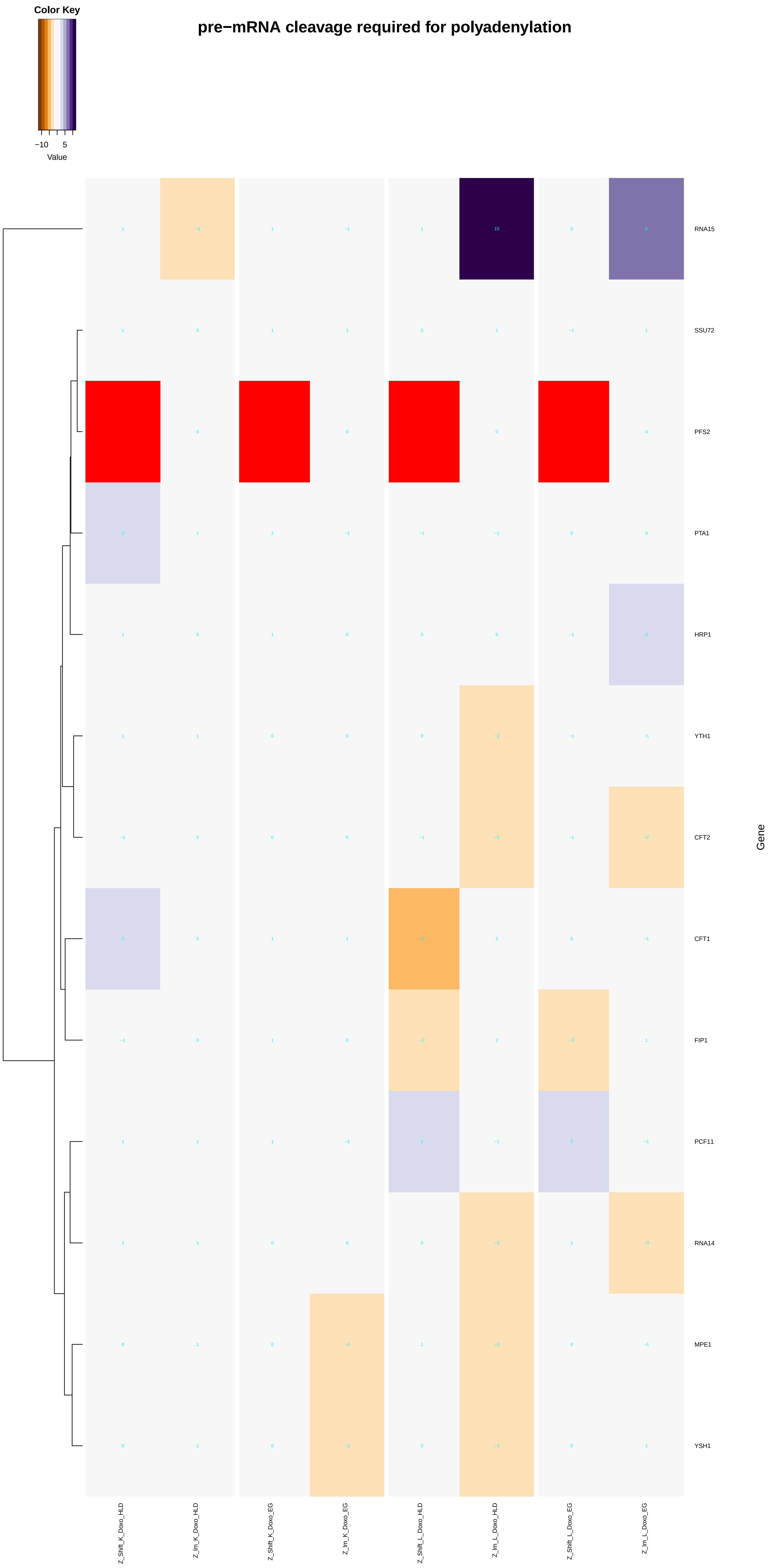

Color Key
−10
5
Value
pre−mRNA cleavage required for polyadenylation
RNA15
SSU72
PFS2
PTA1
HRP1
YTH1
CFT2
CFT1
FIP1
PCF11
RNA14
MPE1
YSH1
Gene
Z_Shift_K_Doxo_HLD
Z_Im_K_Doxo_HLD
Z_Shift_K_Doxo_EG
Z_Im_K_Doxo_EG
Z_Shift_L_Doxo_HLD
Z_Im_L_Doxo_HLD
Z_Shift_L_Doxo_EG
Z_Im_L_Doxo_EG

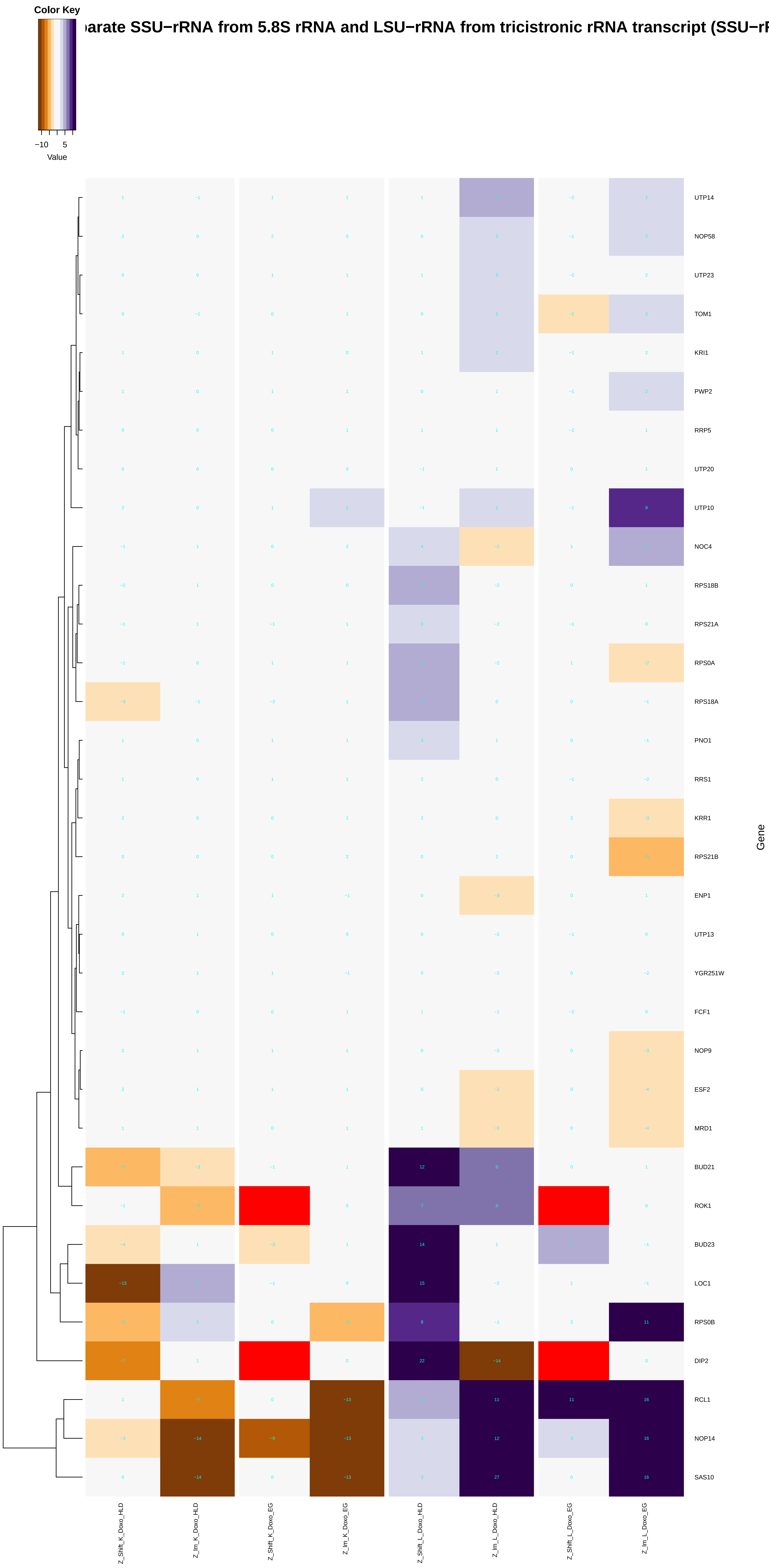

Color Key
−10
5
Value
arate SSU−rRNA from 5.8S rRNA and LSU−rRNA from tricistronic rRNA transcript (SSU−rF
UTP14
NOP58
UTP23
TOM1
KRI1
PWP2
RRP5
UTP20
UTP10
NOC4
RPS18B
RPS21A
RPS0A
RPS18A
PNO1
RRS1
KRR1
RPS21B
ENP1
UTP13
YGR251W
FCF1
NOP9
ESF2
MRD1
BUD21
ROK1
BUD23
LOC1
RPS0B
DIP2
RCL1
NOP14
SAS10
Gene
Z_Shift_K_Doxo_HLD
Z_lm_K_Doxo_HLD
Z_Shift_K_Doxo_EG
Z_lm_K_Doxo_EG
Z_Shift_L_Doxo_HLD
Z_lm_L_Doxo_HLD
Z_Shift_L_Doxo_EG
Z_lm_L_Doxo_EG

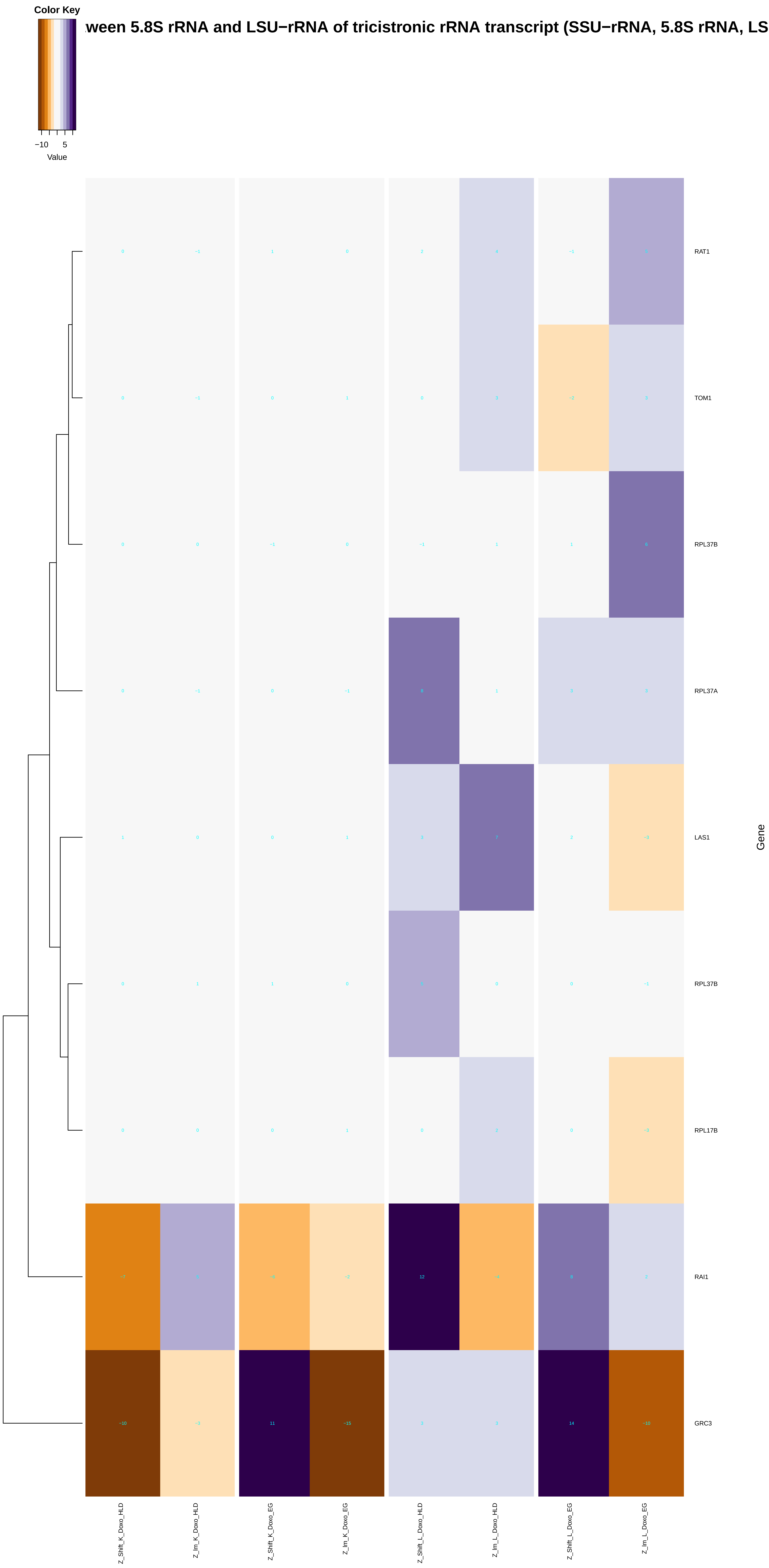
ween 5.8S rRNA and LSU−rRNA of tricistronic rRNA transcript (SSU−rRNA, 5.8S rRNA, LS
Color Key
−10
5
Value
RAT1
TOM1
RPL37B
RPL37A
LAS1
RPL37B
RPL17B
RAI1
GRC3
Gene
Z_Shift_K_Doxo_HLD
Z_Im_K_Doxo_HLD
Z_Shift_K_Doxo_EG
Z_Im_K_Doxo_EG
Z_Shift_L_Doxo_HLD
Z_Im_L_Doxo_HLD
Z_Shift_L_Doxo_EG
Z_Im_L_Doxo_EG

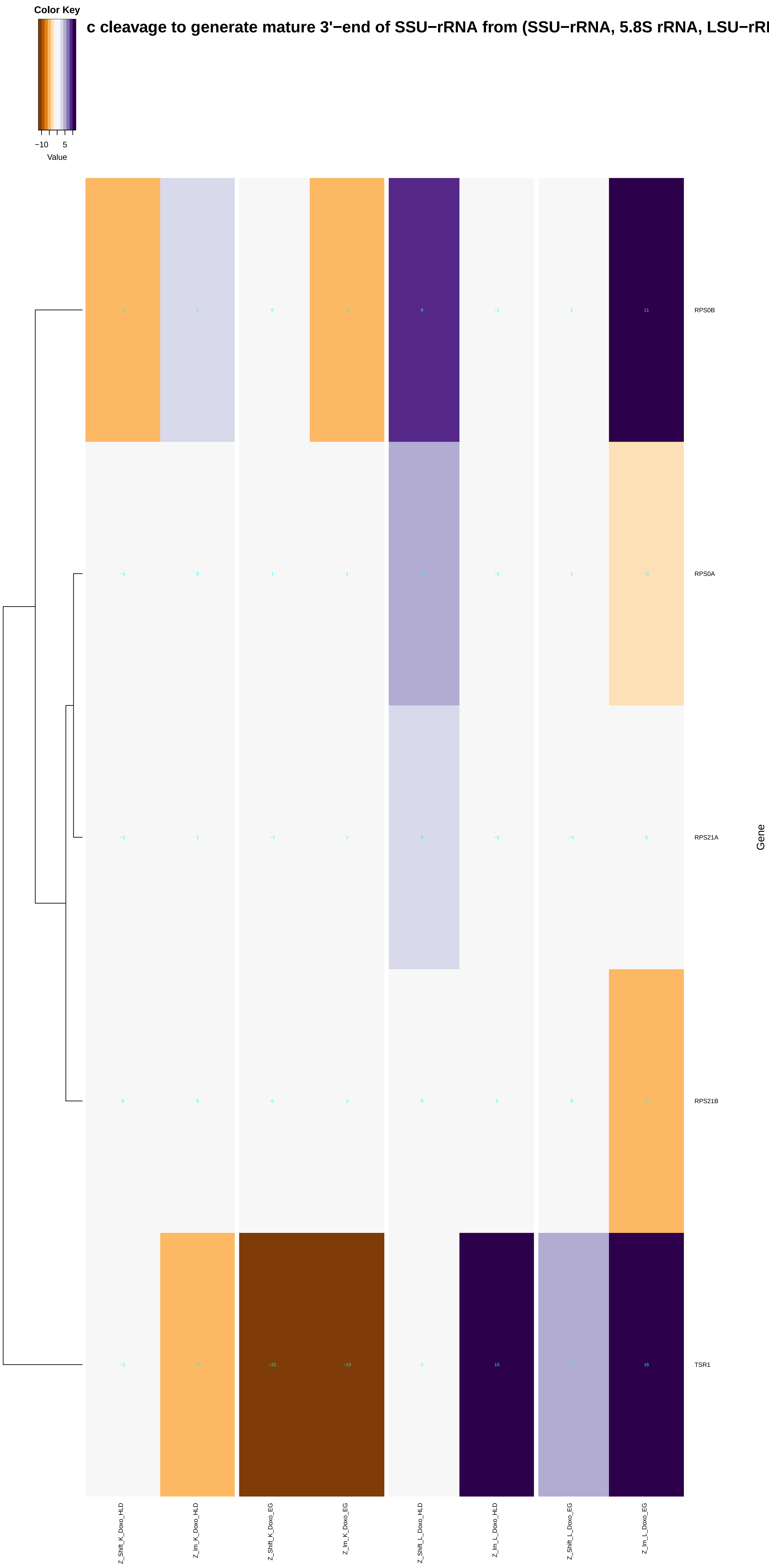
Color Key
−10
5
Value
c cleavage to generate mature 3'−end of SSU−rRNA from (SSU−rRNA, 5.8S rRNA, LSU−rRI
RPS0B
RPS0A
RPS21A
RPS21B
TSR1
Gene
Z_Shift_K_Doxo_HLD
Z_lm_K_Doxo_HLD
Z_Shift_K_Doxo_EG
Z_lm_K_Doxo_EG
Z_Shift_L_Doxo_HLD
Z_lm_L_Doxo_HLD
Z_Shift_L_Doxo_EG
Z_lm_L_Doxo_EG

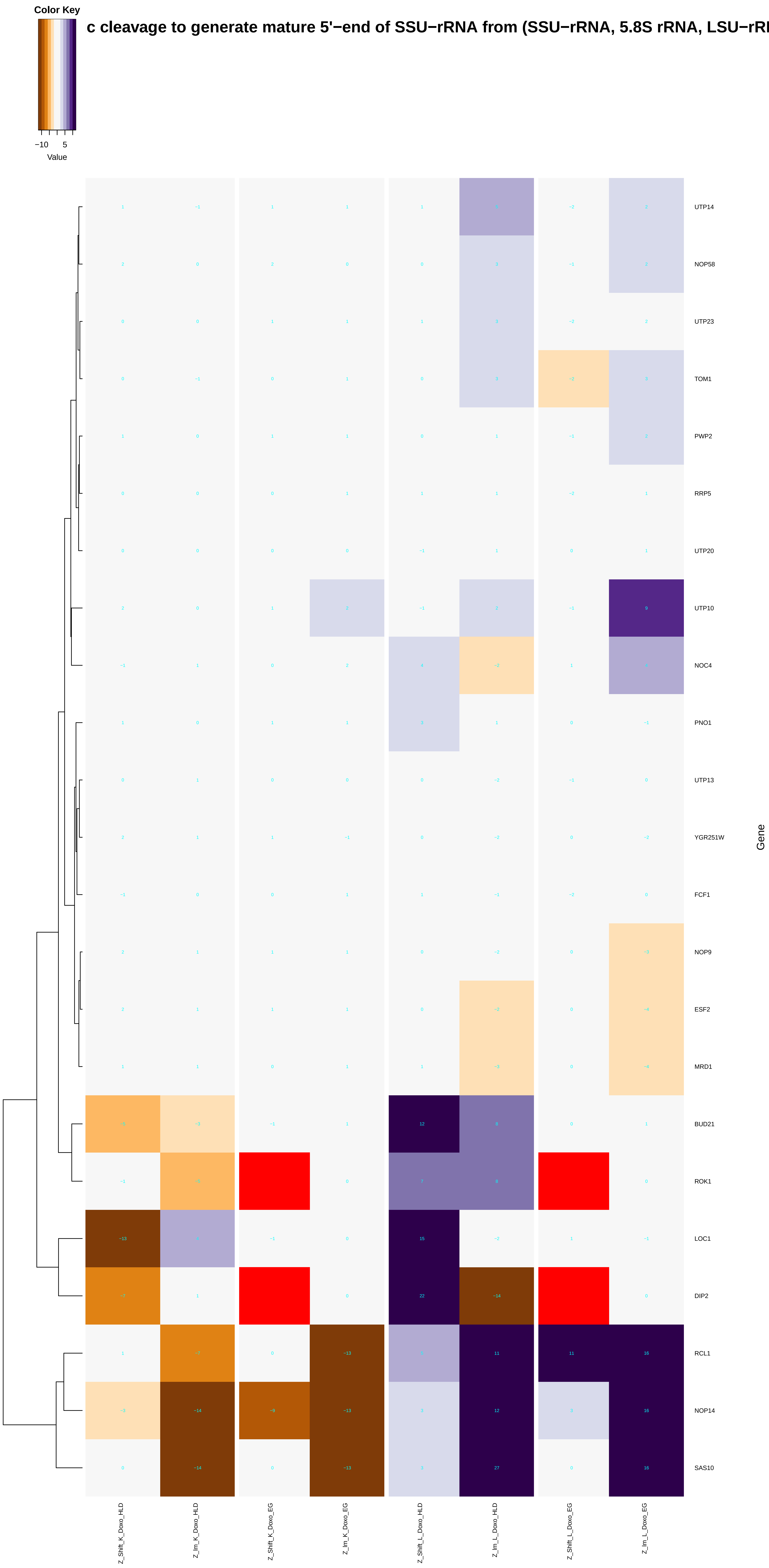
Color Key
−10 5
Value
c cleavage to generate mature 5'−end of SSU−rRNA from (SSU−rRNA, 5.8S rRNA, LSU−rRI
UTP14
NOP58
UTP23
TOM1
PWP2
RRP5
UTP20
UTP10
NOC4
PNO1
UTP13
YGR251W
FCF1
NOP9
ESF2
MRD1
BUD21
ROK1
LOC1
DIP2
RCL1
NOP14
SAS10
Gene
Z_Shift_K_Doxo_HLD
Z_Im_K_Doxo_HLD
Z_Shift_K_Doxo_EG
Z_Im_K_Doxo_EG
Z_Shift_L_Doxo_HLD
Z_Im_L_Doxo_HLD
Z_Shift_L_Doxo_EG
Z_Im_L_Doxo_EG

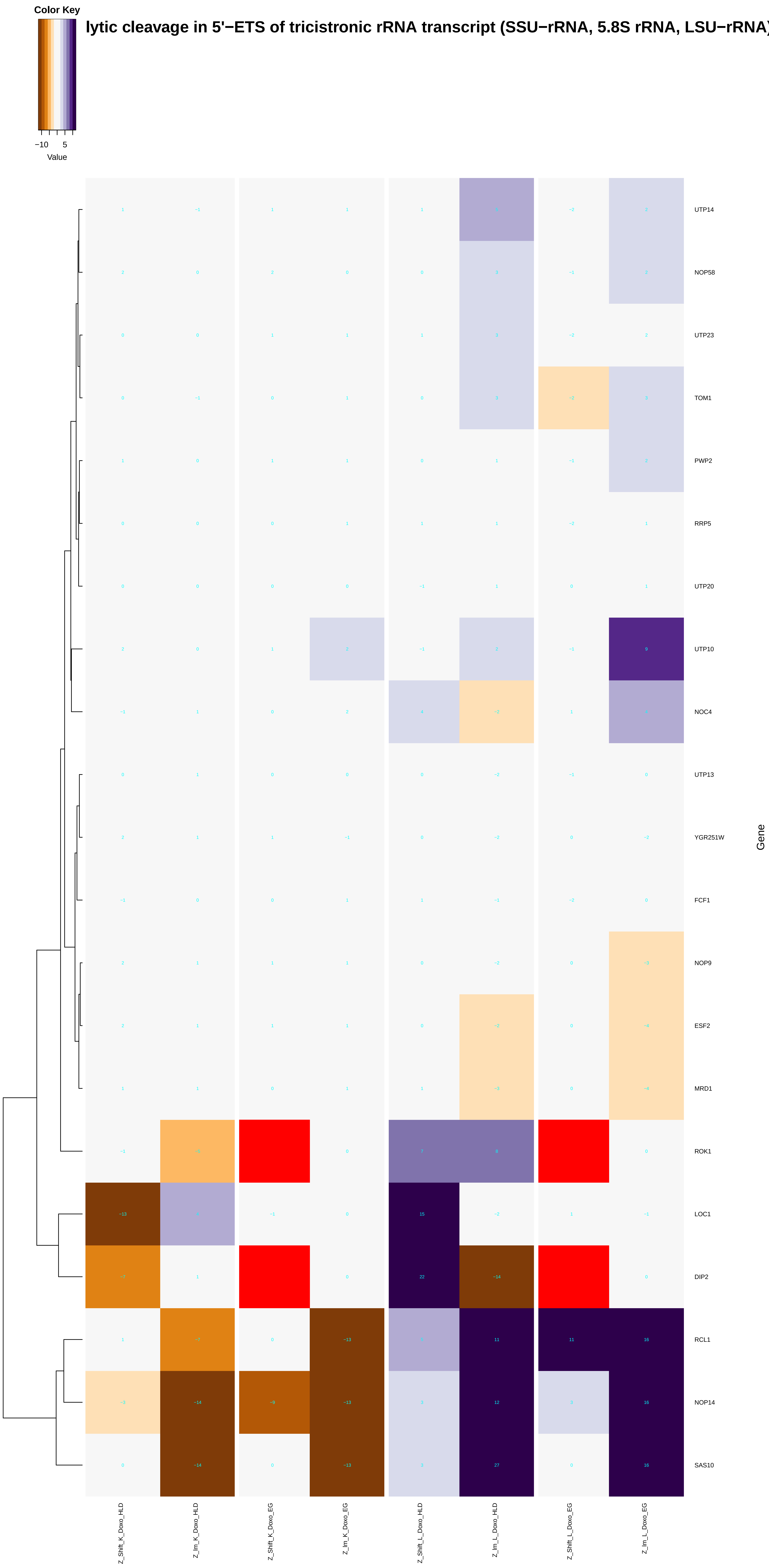
Color Key
−10
5
Value
lytic cleavage in 5'−ETS of tricistronic rRNA transcript (SSU−rRNA, 5.8S rRNA, LSU−rRNA
UTP14
NOP58
UTP23
TOM1
PWP2
RRP5
UTP20
UTP10
NOC4
UTP13
YGR251W
FCF1
NOP9
ESF2
MRD1
ROK1
LOC1
DIP2
RCL1
NOP14
SAS10
Gene
Z_Shift_K_Doxo_HLD
Z_lm_K_Doxo_HLD
Z_Shift_K_Doxo_EG
Z_lm_K_Doxo_EG
Z_Shift_L_Doxo_HLD
Z_lm_L_Doxo_HLD
Z_Shift_L_Doxo_EG
Z_lm_L_Doxo_EG
